# Development of Active Barrier Multilayer Films Based on Electrospun Antimicrobial Hot-Tack Food Waste Derived Poly(3-hydroxybutyrate-*co*-3-hydroxyvalerate) and Cellulose Nanocrystal Interlayers

**DOI:** 10.3390/nano10122356

**Published:** 2020-11-27

**Authors:** Kelly J. Figueroa-Lopez, Sergio Torres-Giner, Inmaculada Angulo, Maria Pardo-Figuerez, Jose Manuel Escuin, Ana Isabel Bourbon, Luis Cabedo, Yuval Nevo, Miguel A. Cerqueira, Jose M. Lagaron

**Affiliations:** 1 Novel Materials and Nanotechnology Group, Institute of Agrochemistry and Food Technology (IATA), CSIC, Calle Catedrático Agustín Escardino Benllonch 7, 46980 Valencia, Spain; kjfigueroal@iata.csic.es (K.J.F.-L.); storresginer@upv.es (S.T.-G.); mpardo@iata.csic.es (M.P.-F.); 2 Gaiker Technological Centre, Department of Plastics and Composites, Parque Tecnológico Edificio 202, 48170 Zamudio, Spain; angulo@gaiker.es; 3 Bioinicia R&D, Bioinicia S.L., Calle Algepser 65, Nave 3, 46980 Paterna, Valencia, Spain; 4 Tecnopackaging S.L., Poligono Industrial Empresarium, Calle Romero 12, 50720 Zaragoza, Spain; josemanuel.escuin@aitiip.co; 5 Food Processing and Nutrition Group, International Iberian Nanotechnology Laboratory (INL), Av. Mestre José Veiga s/n, 4715-330 Braga, Portugal; ana.bourbon@inl.int (A.I.B.); miguel.cerqueira@inl.int (M.A.C.); 6 Polymers and Advanced Materials Group (PIMA), School of Technology and Experimental Sciences, Universitat Jaume I (UJI), Avenida de Vicent Sos Baynat s/n, 12071 Castellón, Spain; lcabedo@uji.es; 7 Melodea Bio-Based Solutions, Faculty of Agriculture-Hebrew University, Rehovot 76100, Israel; yuval@melodea.eu

**Keywords:** PHBV, nanocellulose, multilayers, oregano essential oil, zinc nanoparticles, barrier films, active packaging, migration, cytotoxicity

## Abstract

Active multilayer films based on polyhydroxyalkanoates (PHAs) with and without high barrier coatings of cellulose nanocrystals (CNCs) were herein successfully developed. To this end, an electrospun antimicrobial hot-tack layer made of poly(3-hydroxybutyrate-*co*-3-hydroxyvalerate) (PHBV) derived from cheese whey, a by-product from the dairy industry, was deposited on a previously manufactured blown film of commercial food contact PHA-based resin. A hybrid combination of oregano essential oil (OEO) and zinc oxide nanoparticles (ZnONPs) were incorporated during the electrospinning process into the PHBV nanofibers at 2.5 and 2.25 wt%, respectively, in order to provide antimicrobial properties. A barrier CNC coating was also applied by casting from an aqueous solution of nanocellulose at 2 wt% using a rod at 1m/min. The whole multilayer structure was thereafter assembled in a pilot roll-to-roll laminating system, where the blown PHA-based film was located as the outer layers while the electrospun antimicrobial hot-tack PHBV layer and the barrier CNC coating were placed as interlayers. The resultant multilayer films, having a final thickness in the 130–150 µm range, were characterized to ascertain their potential in biodegradable food packaging. The multilayers showed contact transparency, interlayer adhesion, improved barrier to water and limonene vapors, and intermediate mechanical performance. Moreover, the films presented high antimicrobial and antioxidant activities in both open and closed systems for up to 15 days. Finally, the food safety of the multilayers was assessed by migration and cytotoxicity tests, demonstrating that the films are safe to use in both alcoholic and acid food simulants and they are also not cytotoxic for Caco-2 cells.

## 1. Introduction

Dependence of polymers to fossil fuels and environmental issues related to plastic wastes have motivated the research and development of biodegradable polymer materials based on renewable natural resources. Polyhydroxyalkanoates (PHAs) are one of the most commonly studied biodegradable polyesters that can be obtained by direct biosynthesis using microorganisms and enzymes. In particular, over 300 bacterial species have been reported to accumulate PHAs in their cytoplasm as carbon and energy storage granules [1]. PHAs are homo-, co-, and terpolymers in which their structure is principally constituted by (R)-hydroxy-fatty acids and basically consist of 3-, 4-, 5-, and 6-hydroxycarboxylic acids [2,3]. PHAs can be classified according to the number of carbons in their repeating units. This classification includes short-chain-length PHAs (*scl*-PHAs) with 3 to 5 carbon atoms, which main examples are poly(3-hydroxybutyrate) (PHB) and poly(3-hydroxybutyrate-*co*-3-hydroxyvalerate) (PHBV), showing properties similar to polypropylene (PP), medium-chain-length PHAs (*mcl*-PHAs) with 6 to 14 carbon atoms, such as poly(3-hydroxyhexanoate) (PHHx) and poly(3-hydroxyoctanoate) (PHO), which display elastic properties similar to rubbers and elastomers, and long-chain-length PHAs (*lcl*-PHAs) with more than 14 carbon atoms [3]. The PHB homopolymer and its copolymers with varying ratios of 3-hydroxyvalerate (3HV), that is, PHBV are the most studied PHAs. The inclusion of the 3-HV comonomer reduces the degree of crystallization and crystallization rate of PHB and thus increases its flexibility and reduces its melting temperature (T_m_) [4]. Both the biocompatibility and biodegradability characteristics of PHBV also make this biopolymer an outstanding material with broad applications in different sectors [5]. In this regard, the PHBV films present medium oxygen and water vapor barrier properties, which are relevant characteristics for the design of food packaging articles [6].

Multilayers consist of a sandwich-like structure made up of 12 or more layers typically based on plastics or other materials (e.g., paper) with dissimilar properties glued together where each layer contributes to improve the final properties of the article [7]. Multilayers are typically composed of “structural” and “barrier” layers usually on the outside and inside, respectively. An “active” layer can also be added, either on the outside or inside, depending on the final application. They are obtained by different methods, typically extrusion or solvent casting followed by lamination treatment as well as cast or film extrusion using multiple die designs, where each layer is usually glued by adhesives or “tie” layer resins [8]. In this regard, there are many reports about barrier multilayer systems, which combined hydrophobic biopolymers such as PHBV, polylactide (PLA) or poly(ɛ-caprolactone) (PCL) with hydrophilic ones, such as zein, starch or gelatin, containing nanofillers to improve the mechanical, water vapor, and oxygen barrier properties [8,9,10,11,12]. Likewise, active substances, such as essential oils and metallic nanoparticles, can be incorporated into one of the layers to confer antimicrobial and antioxidant properties to the final multilayer [13].

Cellulose nanocrystal (CNC) has become a new kind of nanofiller for the development of polymer nanocomposites due to its high aspect ratio and specific surface area, high elastic modulus and strength, as well as its non-toxicity, renewability, sustainability, and biocompatibility [14]. Also, it has been reported that CNC acts as a nucleating agent, increasing the polymer crystallization rate and providing increased mechanical properties [15]. CNC can be synthesized from a cellulosic source through isolation mechanisms that are usually attained through top-down methods, such as enzymatic, physical or chemical methodologies for extraction and isolation from crops, plants, and agricultural wastes. Acid hydrolysis of pre-treated (alkaline and bleaching) cellulose is the most commonly used method to isolate amorphous region from crystalline region to obtain CNC [16]. It has a relatively lower aspect ratio with a length of 50–500 nm and a width of 5–70 nm. The nanoparticles derived are pure cellulose and highly crystalline (~88%) with dimensions of ~100–200 nm (length) and cross-sections of ~5–10 nm [17]. CNC exhibits amphiphilic properties due to the high density of hydroxyl groups on their surface and the hydrophobic interactions generated by the crystalline organization along with extensive hydrogen bonding of polymer chains [18].

Among the potential active substances, the most studied ones are oregano essential oil (OEO) and zinc oxide nanoparticles (ZnONPs). Both substances are currently classified as Generally Recognized as Safe (GRAS) by the U.S. Food and Drug Administration (FDA) [19,20]. OEO is obtained by the secondary metabolism of plants and their antimicrobial, antifungal, and antioxidant activities are attributed to its principal volatile compounds, that is, carvacrol, thymol, *p*-cymene, and γ-terpinene [21,22]. ZnONPs are inorganic materials synthesized by mechanochemical processing, sol-gel methods, and spray pyrolysis. Zinc is one of the most used metal in the pharmacy and food industry due to their mechanical and thermal stabilities at ambient temperature, biocompatibility, antimicrobial activity, low cost, and toxicity [23,24]. Hence, the combination of inorganic and organic substances can confer improved active characteristics to the food packaging materials [24,25,26,27].

Electrospinning is an emergent technology with a great deal of potential in food packaging. It can create nano- and micro-scale structures, such as fibers and beads, with variable sizes and porosities of a wide range of polymers [28]. Fibers morphology can be controlled by the properties of the polymer solution, such as polymer type, viscosity, concentration, conductivity, surface tension, and solvent polarity, and also by the processing conditions, for example flow-rate, voltage, and distance to the collector [29]. Furthermore, since the process works at room temperature, it allows the processing of thermolabile substances, which is the case of most essential oils [30]. By the application of mild thermal post-treatments, the electrospinning has shown to be a promising method to obtain monolayer and multilayer systems for food packaging development with functional and active properties [12,22,27,31].

From the above, the aim of this study was to develop active and barrier multilayer films based on PHBV interlayers containing a previously optimized hybrid combination of OEO with ZnONPs and CNC coatings. For this purpose, a PHBV solution containing the active substances was deposited by electrospinning onto a blown film made of a commercial food contact PHA-based resin. The resultant bilayer was then covered on the electrospun layer side by another blown film. A similar structure was also developed including an inner solvent-cast coating of CNC. Both multilayer structures were thereafter laminated by heat in a pilot roll-to-roll laminating system simulating an industrial process and the resultant multilayer films were characterized in terms of their morphological, optical, barrier, and mechanical properties. Furthermore, the antimicrobial activity of the active multilayer film was evaluated against strains of Staphylococcus aureus (*S. aureus*) and Escherichia coli (*E. coli*) foodborne bacteria. Likewise, the antioxidant properties were evaluated in open and closed systems for up to 15 days. Finally, the amount of ZnONPs that might migrate from the active multilayer films into different food simulants and their cytotoxicity for Caco-2 cells were also analyzed.

## 2. Materials and Methods 

### 2.1. Materials

PHBV copolyester was obtained using mixed microbial cultures (MMCs) fed with fermented cheese whey (CW), an industrial residue of the dairy industry, and it was produced by Avecom (Wondelgem, Belgium) following a protocol developed earlier [32]. The content of 3HV in PHBV was 20 mol% determined by gas chromatography (GC) using the method described by Lanham et al. [33] by means of a Bruker 430-GC gas chromatograph equipped with a FID detector and a BR-SWax column (60 m, 0.53 mm internal diameter, 1 mm film thickness, Bruker Optics Inc., Billerica, MA, USA). This PHBV was used to obtain an innovative active hot-tack layer by electrospinning.

A CNC aqueous solution at 2 wt% and a wetting agent at 0.1 wt% were supplied by Melodea Ltd. (Rehovot, Israel). Loctite Liofol PR1550 (primer) was obtained from Henkel Ibérica S.A. (Bilbao, Spain). YPACK210 film for flow-pack application was provided by Tecnopackaging (Zaragoza, Spain). OEO, with a purity >99% and a relative density of 0.925–0.955 g/mL, was obtained from Gran Velada S.L. (Zaragoza, Spain). ZnONPs (CR-4FCC1), 99% purity, specific surface of 4.5 m^2^/g, bulk density of 40 lb/ft^3^, and specific gravity of 5.6 were obtained from GH Chemicals LTD^®®^ (Quebec City, Quebec, Canada). Chloroform, reagent grade with 99.8% purity, and methanol, high-performance liquid chromatography (HPLC) grade with 99.9% purity, were purchased from Panreac S.A. (Barcelona, Spain). Additionally, 1-butanol, reagent grade with 99.5% purity, 2,2-diphenyl-1-picrylhydrazyl radical (DPPH), and (±)-6-hydroxy-2,5,7,8-tetramethylchromane-2-carboxylic acid (Trolox), 97% purity, were purchased from Sigma Aldrich S.A. (Madrid, Spain). Ethanol absolute (≥ 99.9% vol) was supplied by Honeywell^®®^ (Frankfurt, Germany). Acetic acid (AA) glacial (99% purity) was supplied by Fisher Chemical^®®^ (Loughborough, UK). 100% extra virgin olive oil (OO) was provided by Gallo^®®^ (Abrantes, Portugal). Zinc standard for inductively coupled plasma (ICP) calibration (TraceCERT^®®^); 1000 mg/L zinc metal (high-purity quality) in 2% nitric acid (HNO_3_) (prepared with HNO_3_ suitable for trace analysis and high-purity water, 18.2 MΩ.cm, 0.22 µm filtered) and HNO_3_ (70 vol%) were all supplied by Sigma-Aldrich (Darmstadt, Germany). Minimum essential media (MEM) was purchased from Milipore (Berlin, Germany). Trypsin-ethylenediaminetetraacetic acid (EDTA) (0.25% trypsin-0.1% EDTA), penicillin/streptomycin 100x, and fetal bovine serum (FBS) were all bought from Merck Millipore (Burlington, MA, USA). Sodium pyruvate solution 100 mM, resazurin sodium salt, and cell counting kit-8 (CCK-8) were obtained from Sigma-Aldrich (St. Louis, MO, USA).

### 2.2. Food Contact Blown Film

The commercial biodegradable food contact permitted compound YPACK210 was used to make the film. This was supplied by Ocenic Resins S.L. (Valencia, Spain). The compound is based on a commercial PHA, in which the content of PHA is 50 wt%. The film was obtained by film blowing in Tecnopackaging with a blown film extrusion equipment LABTECH LF400 from Techlab Systems S.L. (Lezo, Spain). This machine has the following features: max. bubble diameter 350 mm, variable blowing speed, twin-screw extruder LE25-30/C, large 2.4-meter-high film tower, pneumatically operated film nip rolls, screw speed infinite variable from 0 to 300 rpm, and motorized adjustment of film tower height. The set parameters of the film blowing experiments were: a screw speed of 65 rpm, a screw pressure of 196 bar, a screw temperature profile of 170 °C/170 °C/168 °C/168 °C, a superior roll speed of 1.8 m/min, a collection roll speed of 2.7 m/min, and a tower height of 1500 mm. The resulting blown film had a thickness of around 60 microns and a film width of 250 mm.

### 2.3. CNC Coating

A food contact primer (Loctite LIOFOL PR1550) was applied on a corona discharged (100 watt·cm^2^/min) YPACK210 film. The wetting agent was added into the primer to facilitate the coating of the food contact film surface. The coating trials were conducted, firstly at lab-scale by automatic film applicator and then at larger scale by ROKO (PrintCoat Instruments, Royston, UK). At a lab-scale, the food contact primer with wetting agent was put on the surface treated film by automatic film applicator with a profile rod coater of 6 µm-wet thickness. The primer was dried in oven at 90 °C for 1 min. On top of the primer, the CNC solution was coated using an automatic film applicator: Profile rod coater (100 µm—wet thickness), dry thickness of 2 µm, drying method at 90 °C for 15 min in oven. At large scale, the primer and wetting agent were mixed in an IKA Eurostar 6000 mixer (IKA^®®^-Werke GmbH & Co. KG, Staufen, Germany) at low speed (200 rpm) to avoid bubbles. The food contact primer with wetting agent was put onto the YPACK210 film by a meter bar head with a profile rod of 6 µm in Rotary Koater (ROKO) equipment. Drying was performed at 50 °C. Later, the CNC solution was applied on top of primer with a profile rod of 50 µm. Drying was performed at 90 °C at 1 m/min to ensure complete drying of the CNC solution.

### 2.4. Electrospinning

A neat PHBV solution was prepared by dissolving 8% (wt/vol) in a chloroform/1-butanol 75:25 (vol/vol) mixture at room temperature. A solution containing 2.5 wt% OEO + 2.25 wt% ZnONPs in PHBV was also produced in the same conditions reported previously [21]. The PHBV solutions were electrospun using a roll-to-roll system onto the YPACK210 film by a high-throughput electrospinning/electrospraying equipment Fluidnatek^®®^ LE 500 from Bioinicia S.L. (Valencia, Spain). Both solutions were processed under a constant flow using a 24 emitter multi-nozzle injector with a gauge size of 18, scanning vertically onto the metallic plate. A voltage of 18.5 kV, a flow-rate of 6 mL/h per single emitter, and a tip-to-collector distance of 25 cm were used.

### 2.5. Multilayer Assembly 

**Figure 1:**
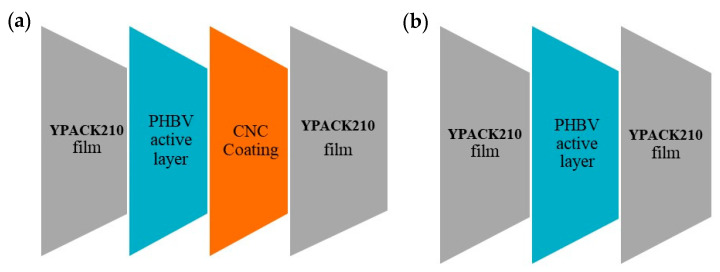
Scheme of the active multilayer films: (**a**) with cellulose nanocrystal (CNC) coating and (**b**) without CNC coating.

Once the active PHBV electrospun hot-tack layer was deposited onto the YPACK210 films with and without the CNC coating, another YPACK210 film was placed as the outer layer according to the scheme displayed in Figure 1. The resulting multilayer structure was assembled using a Reliant lamination equipment with rolls at a speed of 5 m/min at 140 °C during 20 s. The resultant multilayer samples had an average thickness in the 130–150 µm range.

### 2.6. Characterization of the Multilayers 

#### 2.6.1. Film Thickness

Before testing, the thickness of all films was measured using a digital micrometer (S00014, Mitutoyo, Corp., Kawasaki, Japan) with ±0.001 mm accuracy. Measurements were performed and averaged in five different points, two in each end and one in the middle. 

#### 2.6.2. Morphology

The morphology of multilayer films was examined by scanning electron microscopy (SEM). The micrographs were taken using a Hitachi S-4800 electron microscope (Tokyo, Japan) at an accelerating voltage of 10 kV and a working distance of 8–10 mm. The samples were previously sputtered with a gold-palladium mixture for 3 min under vacuum. The average fiber diameter was determined via the ImageJ software v 1.41 using at least 20 SEM images.

#### 2.6.3. Transparency

The light transmission of the YPACK210 film and active multilayer films was determined in specimens of 50 mm × 30 mm by quantifying the absorption of light at wavelengths between 200 nm and 700 nm, using an UV–Vis spectrophotometer VIS3000 from Dinko, Instruments (Barcelona, Spain). The transparency value (T) was calculated using the following Equation (1):

(1) $$\;\;\;\;\;\;T=\frac{A_{600}}{L}$$

where and $A_{600}$ is the absorbance at 600 nm, and *L* is the film thickness (mm).

#### 2.6.4. Color

The color of the YPACK210 film and multilayer films was measured by using a Chroma Meter CR-400 (Konica Minolta, Tokyo, Japan) with illuminant D65. The color difference (∆E*) was calculated by using the following Equation (2):

(2) $$\Delta E={\left[{\left(\Delta L^{*}\right)}^{2}+{\left(\Delta a^{*}\right)}^{2}+{\left(\Delta b^{*}\right)}^{2}\right]}^{0.5}$$

where ∆E*, ∆a* and ∆b* corresponded to the differences between the color parameters of multilayer films containing OEO and ZnONPs and the values of the reference film (neat blend) (a* = 0.74, b* = −0.41, L* = 90.44).

#### 2.6.5. Water Vapor Permeance 

The water vapor permeance (WVP) of the multilayer films was determined according to the ASTM gravimetric method using Payne permeability cups (Elcometer SPRL, Hermelle/s, Lieja, Belgium) of 3.5 cm diameter. One side of the films was exposed to 100% relative humidity (RH) by avoiding direct contact with liquid water. Then the cups containing the films were secured with silicon rings and stored in a desiccator at 25 °C and 0% RH. The control samples were cups with aluminum films to estimate the solvent loss through the sealing and samples placed in cups but without permeant. The cups were weighed periodically after the steady state was reached. Measurements were done in triplicate for each type of samples. WVP was calculated from the steady-state permeation slopes obtained from the regression analysis of weight loss data over time.

#### 2.6.6. Limonene Vapor Permeance

Limonene permeance (LP) vapor was measured as described above for WVP. For this, 5 mL of _D_-limonene was placed inside the Payne permeability cups. The test was performed at 25 °C and 40% RH. The control samples were cups with aluminum films to estimate the solvent loss through the sealing and samples placed in cups but without permeant to account for weight changes due to moisture sorption.

#### 2.6.7. Mechanical Tests

Tensile tests were conducted in a universal testing machine Shimatzu AGS-X 500N (Shimatzu, Kyoto, Japan) at room temperature with a cross-head speed of 10 mm/min. Dumbbell film samples according to ASTM D638 (Type IV) standard were die-cut from the multilayer assembly both in machine direction (MD) and in the transversal direction (TD). All the samples were stored in a vacuum desiccator at room temperature until tested. At least six samples were tested for each multilayer. The average values and standard deviations of the mechanical parameters were reported.

#### 2.6.8. Antimicrobial Tests 

*S. aureus* CECT240 (ATCC 6538p) and *E. coli* CECT434 (ATCC 25922) strains were obtained from the Spanish Type Culture Collection (CECT, Valencia, Spain) and stored in phosphate buffered saline (PBS) with 10 wt.% tryptic soy broth (TSB, Conda Laboratories, Madrid, Spain) and 10 wt% glycerol at −80 °C. Previous to each study, a loopful of bacteria was transferred to 10 mL of TSB and incubated at 37 °C for 24 h. A 100-µL aliquot from the culture was again transferred to TSB and grown at 37 °C to the mid-exponential phase of growth. The approximate count of 5 × 10^5^ colony forming units (CFU)/mL of culture having absorbance value of 0.20 as determined by optical density at 600 nm (Agilent 8453 UV–visible spectrum system, Agilent Technologies Deutschland GmbH, Waldbronn, Germany).

The antimicrobial performance of the active multilayer films was evaluated by using a modification of the Japanese Industrial Standard JIS Z2801 (ISO 22196:2007) [12]. A microorganism suspension of *S. aureus* and *E. coli* was applied onto the active multilayer films containing OEO and ZnONPs with CNC (measured by both sides) and without CNC, and in the multilayers without OEO and ZnONPs (as negative control). The samples, sizing 1.5 cm × 1.5 cm, were placed in either hermetically closed or open bottles, the here so-called closed and open systems. After incubation at 24 °C at least 95% RH for 24 h, bacteria were recovered with PBS, 10-fold serially diluted, and incubated at 37 °C for 24 h in order to quantify the number of viable bacteria by conventional plate count. The antimicrobial activity was evaluated from 1 (initial day), 3, 8, and 15 days. The value of the antimicrobial activity (*R*) was calculated as follows:

(3) $$R=\left[Log\left(\frac{B}{A}\right)-Log\left(\frac{C}{A}\right)\right]=Log\left(\frac{B}{C}\right)$$

where *A* is the average of the number of viable bacteria on the control sample immediately after inoculation, *B* is the average of the number of viable bacteria on the control sample after 24 h, and *C* is the average of the number of viable bacteria on the test sample after 24 h. Three replicate experiments were performed for each sample and the antibacterial activity was evaluated with the following assessment: Nonsignificant (*R* < 0.5), slight (*R* ≥ 0.5 and <1), significant (*R* ≥ 1 and <3), and strong (*R* ≥ 3) [34].

#### 2.6.9. Antioxidant Measurements

The antioxidant activity of the multilayer films was determined following the DPPH assay. The film samples were stored for 1 (initial day), 3, 8, and 15 days in both open and closed systems. In each measurement, approximately 0.1 g of film was weighed in triplicate in cap vials and then an aliquot of 10 mL of a stock DPPH solution (0.05 g/L in aqueous methanol 80 vol%) was added. Vials without samples were also prepared as controls. The blank was the sample in aqueous methanol 80 vol% without DPPH. All the samples were prepared and immediately stored at room temperature for 2 h in darkness. After this, the absorbance of the solution was measured at 517 nm in the UV 4000 spectrophotometer from Dinko Instruments (Barcelona, Spain). Results were expressed as the percentage of inhibition to DPPH following Equation (4) [35] and μg equivalent of Trolox per gram of sample, employing a previously prepared calibration curve of Trolox (0–1000 µM).

(4) $$Inhibition\;DPPH\;\left(\%\right)=\;\frac{A_{Control}-\left(A_{sample}-A_{blank}\right)}{A_{control}}*100$$

where $A_{control},\;A_{blank},\;\mathrm{and}\;A_{sample}$ are the absorbance values of the DPPH solution, methanol with the test sample, and the test sample, respectively.

#### 2.6.10. Migration Tests

The specific migration test conditions were followed by the European Normative EC 13130-1:2004 [36]. For this purpose, the Inductively Coupled Plasma-Optical Emission Spectroscopy (ICP-OES) was used to determine the migration of ZnONPs particles from the active multilayer films using standardized cell (MigraCell^®®^, FABES Forschungs-GmbH^®®^, Munich, Germany) for overall/specific migration with an area of 0.75 dm^2^ in three food simulants that were sealed in clean wide-mouth jars. These simulant systems were ethanol (83.33 mL of 10% vol/vol at 40 °C for 10 days), acetic acid (83.33 mL of 3% wt/vol at 40 °C for 10 days), and olive oil (83.33 g of 100% at 10 °C for 10 days). For each formulation, the specific migration tests were carried out in triplicate. As procedural blanks, the food simulant was filled into sealed jars and stored under the same conditions to check for contamination. All results were blank subtracted. After the incubation period, the multilayer films containing OEO/ZnONPs were removed, whereas the ethanol and acetic acid simulants were evaporated on an electric hot plate (100–150 °C) and subsequently digested with 1.2 mL of 70 vol% HNO_3_. After the digestion process, the samples were resuspended in a 10 vol% acidic solution. The simulant olive oil (300 mg) was mixed with 10 mL HNO_3_ (70 vol%) and 2 mL of hydrogen peroxide (30 vol%) and thus digested in microwave. After the digestion process, 1 mL of sample was resuspended in a 2 vol% acidic solution. All digested sampled were introduced for metal quantification by Inductively Coupled Plasma-Optical Emission Spectrometry (ICP-OES) using a Spectrometer ICPE-9000 (Shimadzu^®®^, Tokyo, Japan) equipped with axial torch, an ultrasonic nebulizer for higher sensitivity, and a charge-coupled device (CCD) detector. The instrumental parameters employed for ICP-OES analysis were: nebulizer gas flow (0.70 L/min Ar); auxiliary gas flow (0.60 L/min Ar); Plasma (10 L/min Ar); Ar Gas P (478.66 kPa); ICP RF power (1.20 kW); direction: axial; rotation speed (20 rpm); CCD Temp (−15 °C); vacuum level (6.9 Pa). The linearity of the calibration curve was considered acceptable for a correlation coefficient of *R*^2^ > 0.999.

#### 2.6.11. Cytotoxicity Assay

Caco-2 cells, clone HTB-37™, from human colon carcinoma, were obtained from the American Type Culture Collection (ATCC^®®^ Manassas, Virginia, USA). Caco-2 cells (passage 25-40) were cultured in minimum essential medium (MEM), supplemented with 20% FBS, 1% sodium pyruvate, and 1% penicillin/streptomycin. The cells were kept at 37 °C and 5% CO_2_ in 75 cm^2^ flasks. For the cytotoxicity assessment, confluent cells were detached using 0.25% trypsin-EDTA solution, then precipitated by centrifugation at 1080 rpm for 5 min and resuspended in fresh medium MEM at a concentration of 1 × 10^5^ cells/mL. Cells were seeded onto 96-wells plates at a density of 1 × 10^4^ cells (100 µL of cellular suspension) per well and left adhering overnight in a humidified atmosphere of 5% CO_2_ in air at 37 °C.

The cytotoxicity was indirectly determined by CCK-8 and resazurin assays. The evaluation was performed through indirect contact with Caco-2 cells. After cells adhesion, the culture medium was removed and replaced by 200 µL of culture medium. The active multilayer films with and without CNC (28 mm^2^) were placed onto the top of the culture medium, above the cells surface and incubated for 24 or 48 h. At each time-point, the multilayer films were removed and replaced by the biomarker.

##### CCK-8 Assay

CCK-8 is a colorimetric assay, which uses a highly water-soluble tetrazolium salt, exhibiting superior detection sensitivity than other tetrazolium salts-based assays [37]. In the CCK-8 measurement, the dye WST-8 [2-(2-methoxy-4-nitrophenyl)-3-(4-nitrophenyl)-5-(2,4disulfophenyl)-2H-tetrazolium, monosodium salt] is reduced by dehydrogenase in cells to form a water-soluble orange-colored product (formazan). The amount of formazan dye produced is directly correlated with the number of living cells. 100 µL of CCK-8, diluted at 5% (vol/vol) in culture medium, were added to each well. After 3 h of incubation, the absorbance was measured at 450 nm using a Microplate Reader (Synergy, BioteK H1, BioTek Instruments, Winooski, VT, USA). The cell viability was expressed in percentage of absorbance in treated cells in relation to the absorbance of cells growing in MEM. A negative control was performed using cells growing in culture medium (MEM), considered as 100% cell viability. A positive control was done using 10 vol% DMSO.

##### Resazurin Assay

Resazurin dye is a cell permeable redox indicator that has been broadly used as an indicator of cell viability in proliferation and cytotoxicity assays [38]. Viable cells with active metabolism can reduce resazurin into the resorufin product, which is pink and fluorescent. The quantity of resorufin produced is proportional to the number of viable cells. After adhesion, the culture medium was removed and replaced by culture medium with 0.01 mg/mL resazurin. Multilayer film samples of 0.4 mm × 0.4 mm were added to each well and incubated for 24 h and 48 h. A negative control was performed using the cells growing in the culture medium (considered as 100% cell viability) and 40% (vol/vol) DMSO was used as a positive control (cell death).

The fluorescence intensity, which is proportional to the cell viability, was directly measured at each time point (24 and 48 h) using a Microplate Fluorescence Reader from BioTek Instruments at an excitation wavelength of 560 nm and an emission wavelength of 590 nm. The percentage of cell viability was expressed as fluorescence of treated cells compared to the fluorescence of cells growing in the culture medium as follows:

(5) $$\mathrm{Cell}\;\mathrm{viability}\;(\%)=\frac{\left(F_{TC}-F_{S}\right)}{\left(F_{C}-F_{CM}\right)}\times 100$$

where *F_TC_* is the fluorescence of treated cells, *F_S_* is the fluorescence of the sample in the culture medium (without cells), *F_C_* is the fluorescence of cells growing in the culture medium, and *F_CM_* is the fluorescence of culture medium (without cells).

### 2.7. Statistical Analysis

The color, transparency, barrier properties, and antioxidant activity were evaluated through analysis of variance (ANOVA) with 95% significance level (*p* ≤ 0.05) and a multiple comparison test (Tukey) to identify significant differences among the treatments. Each treatment was done in triplicate. For this purpose, the software OriginPro8 (OriginLab Corporation, Northampton, MA, USA) was used.

## 3. Results and Discussion

### 3.1. Morphology

**Figure 2:**
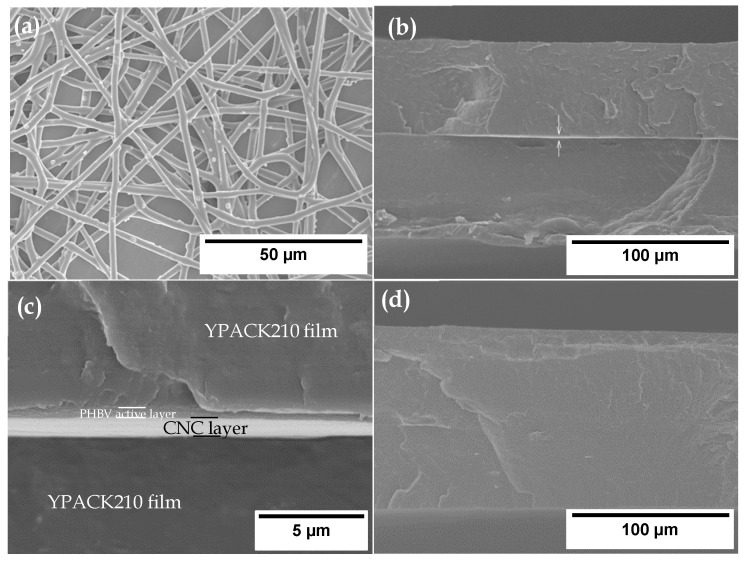
Scanning electron microscopy (SEM) micrographs of (**a**) the electrospun fibers of the cheese whey (CW) derived poly(3-hydroxybutyrate-*co*-3-hydroxyvalerate) (PHBV) containing 2.5 wt% oregano essential oil (OEO) + 2.25 wt% zinc oxide nanoparticles (ZnONPs) and of the active multilayer films (**b**,**c**) with cellulose nanocrystal (CNC) coating and (**d**) without CNC coating. Images taken at 800×, 500×, and 6000× show scale markers of 50, 100, and 5 μm, respectively.

The morphologies of the electrospun CW derived PHBV fibers and the multilayer films were analyzed by SEM in Figure 2. One can observe in Figure 2a that electrospinning yielded fibers with a smooth surface and free of beaded regions, having a final thickness for the layer of approximately 0.6 μm. The thickness of the multilayer films with CNC and without CNC was 140 and 131 μm, respectively. This by no means implies that the difference in thickness is accounted for by the layer of CNC, but that the substrate film used had variations along the width generated during the film blowing process. The most representative micrographs of each multilayer in their cross-sections are also shown in Figure 2. On the one hand, the multilayer film with CNC, included in Figure 2b, showed clearly the presence of the CNC coating. A higher magnification of this cross-section, shown in Figure 2c, revealed that the thickness of the resultant CNC interlayer was approximately 1 μm, and also the presence of the electrospun hot-tack active layer. It can also be observed that both interlayers were well adhered in the multilayer structure, suggesting good adhesion with the YPACK210 film. On the other hand, the active multilayer film without CNC, shown in Figure 2d, also presented a homogeneous and continuous cross-section. However, individual layers cannot be discerned, which can be attributed to the good adhesion between the electrospun hot-tack layer and YPACK210 film since both are based on PHAs. As previously researched, the electrospun interlayers have the capacity to act as a tie layer after a mild annealing post-processing step due to their high porosity and large surface-to-volume ratio, which avoid the need for adhesives or tie resins that are usually employed in conventional processes to develop multilayer systems [39,40]. Similar results were reported by Fabra et al. [41] about multilayers consisted of PHBV films obtained by both solution casting and compression molding that were coated with zein ultrathin fiber mats by electrospinning. The last ones presented a homogeneous and completely smooth surface after being annealed in a hot press. Similarly, Cherpinski et al. [42] developed multilayer films based on PHB nanopapers, showing that the multilayers presented a similar continuous structure and strong interlayer adhesion, which was attributed to the coalescence process at the fiber interphase. This annealing process, performed at temperatures well below the polymer’s melting point, has been recently described and better understood by Melendez-Rodriguez et al. [43]

### 3.2. Transparency and Color

**Figure 3:**
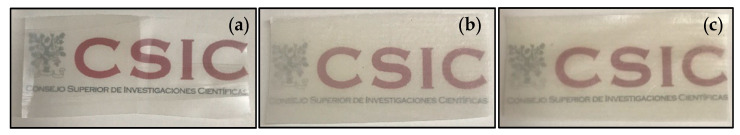
Visual aspect of: (**a**) YPACK210 film; (**b**) active multilayer films with cellulose nanocrystal (CNC) coating; (**c**) active multilayer films without CNC coating. Films are 5 cm × 2 cm.

Visual aspect of the active multilayer films is presented in Figure 3 and the transparency values of the films are shown in Table 1. This parameter is directly related to the surface and the internal structure of the material that is known to influence the light transmission and dispersion, thus affecting the transparency/opacity ratio [9]. The neat blown YPACK210 film, which is shown as reference in Figure 3a, presented the highest transparency value, that is, 6.83 ± 0.12. The active multilayer film with CNC showed a lower value transparency of 4.29 ± 0.15, whereas the active multilayer film without CNC presented a value of 5.94 ± 0.17. Therefore, lower transparency values were attained in the multilayer films that can ascribed to the fact that the interlayers were made of different materials that influenced on the light transmission and dispersion. In this regard, Cerqueira et al. [44] also showed that the presence of different outer layers based on PHBV and a zein interlayer with or without cinnamaldehyde led to a reduction in the transparency of the resultant multilayer films. In our previous report [24], the optical properties of similar active monolayer films based on PHBV prepared by electrospinning presented a reduction in transparency, which was mainly ascribed to the presence of both OEO and ZnONPs. Fabra et al. [45] also reported differences in the internal transmittance values of multilayer films based on thermoplastic corn starch (TPCS) containing bacterial cellulose nanowhiskers (BCNWs) prepared by melt mixing and coated with PHB fibers by electrospinning. The reduction in transparency was attributed to the presence and different degree of dispersion of BCNWs, resulting in a different refractive index. However, it is also worthy to mention that in the case of the here-developed multilayers based on CNC and electrospun PHBV interlayers, a notable transparency was still attained. Moreover, the effect of the CNC coating on the multilayer transparency was relatively low due to the low thickness and high homogeneity of this nanostructure cellulosic material. From a point of view of food preservation, the lower transparency in the multilayer films can also be positive due to their higher barrier capacity to block light, by which the photo-oxidation of organic compounds and degradation of vitamins and pigments of the food products can be reduced [46].

The color parameters of active multilayer films were evaluated through CIELab* coordinates (L*, a*, b*), which represent the human visual color scale, and they were also reported in Table 1. It can be observed that multilayer films developed a slightly higher yellow appearance than the reference sample YPACK210 film, which probably originated by thickness doubling and also the presence of OEO since essential oils are known to increase yellow levels [21]. Accordingly, the values of a* coordinate, which represents the red and green colors, were 0.25–0.28 whereas the b* coordinate, which corresponds to yellow and blue colors, presented values of 1.31–1.42, and luminosity or brightness, represented by L*, was in the range of 89.81–89.86. Furthermore, one can also observe that the incorporation of the CNC coating in the form of interlayer into the active multilayer films did not show significantly differences in CIELab* coordinates. In particular, the Δ*E* values of the active multilayer films, when referenced to the YPACK210 film, were 1.88 (with CNC) and 1.84 (without CNC). In general terms, the color changes were based on a decrease in L* and an increase in the b* coordinate, which were responsible for the increase in the yellow tone of the active multilayer films. A similar yellowing was previously observed by Melendez-Rodriguez et al. [47], who incorporated eugenol encapsulated in silica into PHBV by electrospinning. This effect was ascribed to the intrinsic eugenol color, which is a yellow oily liquid.

### 3.3. Barrier Properties

In general, biopolyesters show lower water barrier properties than conventional petrochemical polymers such as polyethylene terephthalate (PET), which is detrimental for the development of packaging materials for food preservation [48]. One of the advantages of multilayer systems is the improvement of the barrier characteristics due to the use of barrier materials typically in the inner layers and protected from moisture and mechanical stress by the structural layers [49]. Permeance values in terms of water and limonene vapors, that is WVP and LP, of the active multilayer films are shown in Table 2. The WVP value (0.87 × 10^−11^ kg·m^−2^·Pa^−1^·s^−1^) and LP value (1.36 × 10^−11^ kg·m^−2^·Pa^−1^·s^−1^) of the active multilayer films with CNC were lower than the neat YPACK210 film modeled for similar thickness, which respectively presented a WVP value of 1.38 × 10^−11^ kg·m^−2^·Pa^−1^·s^−1^ and a LP value of 1.62 × 10^−11^ kg·m^−2^·Pa^−1·^s^−1^. Alternatively, the active multilayer film without CNC showed values of WVP and LP values of 1.32 × 10^−11^ kg·m^−2^·Pa^−1^·s^−1^ and 1.59 × 10^−11^ kg·m^−2^·Pa^−1^·s^−1^, respectively, being also higher than those attained for the multilayer with CNC. This improvement in water and _D_-limonene barrier properties is ascribed primarily to the presence of the CNC thin coating, which is known to improve above all oxygen [31,42]. In addition, the presence of the active electrospun PHA layer containing OEO and ZnONPs, which are hydrophobic substances, may also contribute to block the diffusion of water molecules through the material [31]. Oxygen permeability could not be measured since the release the essential oil during the testing could alter the reading of the tester and, according to the manufacturer, could even damage the sensor. However, CNC coatings have been tested in other multilayers, yielding very strong oxygen permeance reductions, of more than 89% decrease when protected from moisture [42,50,51].

Other works have also reported important improvements in barrier performance by means of electrospun multilayers. For instance, Fabra et al. [52] compared the effect of adding different interlayers of electrospun whey protein isolate (WPI), pullulan, and zein to a PHA film obtained by compression molding. The addition of both electrospun zein and pullulan nanofibers improved the water vapor permeability by 28–35%. In another study, Cherpinski et al. [42] improved the barrier properties of electrospun PHB and PHBV double side coatings by using cellulose nanofibrils (CNFs) and lignocellulose nanofibrils (LCNFs) interlayers. Wang et al. [53] also reported that the water vapor value of the multilayer film obtained by electrospinning with ethylcellulose nanofibers, as the outer layer, and curcumin-loaded gelatin nanofibers, as the inner one, was significantly lower (4.68 × 10^−^^12^ g·cm·cm^−^^2^·s^−^^1^·Pa^−^^1^) than the equivalent gelatin film with or without curcumin (5.45 × 10^−^^12^ g·cm·cm^−^^2^·s^−^^1^·Pa^−^^1^). The active multilayer films developed herein also showed slightly improved barrier properties.

### 3.4. Mechanical Properties

The mechanical properties in terms of elastic modulus (E), tensile strength at break (σ_b_), elongation at break (ε_b_), and static toughness (T) of the YPACK210 film and active multilayer films in transversal direction (TD) and machine direction (MD) are gathered in Table 3. The YPACK210 film presented an E value of 2066 MPa (TD) and 2510 MPa (MD), a σ_b_ value of 23.1 MPa (TD) and 29.6 MPa (MD), and a ɛ_b_ value of 173% (TD) and 76.3% (MD). The difference in the mechanical behavior between the two directions is ascribed to the orientation of the biopolymer molecules during manufacturing of the YPACK210 film.

It can be observed that all the mechanical parameters decreased for the multilayer systems. Hence, the E values for the multilayer without CNC dropped by approximately 30% for both TD and MD samples, presenting values of 1491 MPa (TD) and 1828 MPa (MD), respectively. Similarly, the σ_b_ values of the multilayer were lower than the monolayer YPACK210 film, showing a decrease close to 20% in both tested directions. Therefore, the assembly of the multilayer system resulted in a decrease in the mechanical performance in comparison to the YPACK210 films. The lower mechanical resistance of the multilayer films can be ascribed to a delamination failure, which indicates that the interlayer adhesion was weaker, as expected, than the mechanical strength of the neat YPACK210 film.

### 3.5. Antimicrobial Activity

In food packaging, antimicrobial properties are indispensable to avoid or delay the microbiological reactions of the food products [54]. In this context, different natural substances, which is the case of essential oils and metallic nanoparticles, show the capacity to inhibit the growth of Gram positive (G+) and Gram negative (G−) bacteria. The antimicrobial performance of the active multilayer films with and without CNC was evaluated in an open and closed systems against *S. aureus* (+) and *E. coli* (G−) strains for 1, 3, 8, and 15 days. Table 4 shows the *S. aureus* and *E. coli* reduction values, that is, R, of the active multilayer films in an open system. The active multilayer film with CNC, measured by the active side after 15 days, showed *S. aureus* and *E. coli* reduction values of 1.33 and 1.26, respectively, which correspond to a significant reduction of the bacterial growth (*R* ≥ 1 and <3). While the active multilayer film with CNC, measured by the CNC side, showed a slightly lower reduction against both bacteria, it was still significant. At 15 days, reduction value against *S. aureus* and *E. coli* of 1.08 and 1.04 were respectively attained. The slightly difference with the active multilayer film measured by the active side is due to presence of the high barrier CNC coating, which could promote a slow release of the active compounds to the film surface. On the other hand, the active multilayer film without CNC reached, at 15 days, a slightly increase in the both bacteria reduction due to the improved release of the active compounds. The reduction of *S. aureus* was 1.35 and *E. coli* was 1.27, which corresponds to a significant reduction (*R* ≥ 1 and <3).

Table 5 presents the *S. aureus* and *E. coli* reduction values of the active multilayer films in the so-called closed system, which better resembles an actual packaging system. It was observed a slight difference in the reduction values reached at 15 days for the active multilayer films with CNC and without CNC against both bacteria when compared to the results obtained in the open system. This slightly increment can be associated to the release of the volatile compounds of OEO, which could accumulate in the headspace during the antimicrobial measurements and increased its concentration on the multilayer film surface. This phenomenon was observed in another research work using essential oils and natural extracts, suggesting that the use of these active volatile substances can be very relevant for food packaging [21,22]. Although the evaluated multilayer films presented a significant inhibition (*R* ≥ 1 and <3), it was observed that the presence of CNC into one of the interlayers yield to sustain release of the active compounds. Besides that, in our previous study it was reported the optimization of the electrospun monolayer film containing 2.5 wt% OEO + 2.25 wt% ZnONPs [24], which showed a strong reduction (*R* ≥ 3) against *S. aureus* and *E. coli*., reason by which it was selected to develop the present multilayer films.

From the above, it is clearly seen that the antimicrobial activity of the monolayer films decreases in multilayer systems. However, the multilayers films obtained in this research presented a significant reduction against both G+ and G− bacteria, which enhances the application of this biodegradable active materials for food packaging applications. Other authors have also reported multilayer systems with antimicrobial performance. In this way, Lee et al. [55] developed a multilayer film based on polypropylene (PP), PET, and low-density polyethylene (LDPE) containing star anise essential oil and thymol coating layers by bar coating and adhesive lamination processes. Authors concluded that the developed multilayer can show potential applicability as an active food packaging material with insect repellent and antimicrobial activities. In another study, Gherardi et al. [56] evaluated an antimicrobial packaging based on a multilayer commercial material composed of polyester, aluminum, and polyethylene joined by two adhesive layers and containing cinnamon essential oil. The resultant multilayer demonstrated a high antimicrobial activity in both inoculated culture media (in vitro) and tomato puree (in vivo) when packaged within the multilayer material against *E. coli* O157:H7 and *Saccharomyces cereviase* (*S. cereviase)* measured after 24 h and 48 h, respectively. Cerisuelo et al. [57] also developed and successfully tested the antimicrobial activity of a multilayer material based on PP and PET coated with poly(ethylene-*co*-vinyl alcohol) (EVOH) that contained carvacrol, citral, marjoram essential oil or cinnamon bark essential oil through the application of a corona discharge followed by a polyethyleneimine (PEI)-based primer. In addition, Cerqueira et al. [44] studied the antimicrobial activity of multilayer films using PHBV as support and zein interlayer with or without cinnamaldehyde directly electrospun onto one side of the PHBV film and using a PHBV film as the outer layer. This active multilayer system showed a greater antibacterial activity against *Listeria monocytogenes* (*L. monocytogenes*).

### 3.6. Antioxidant Activity

**Figure 4:**
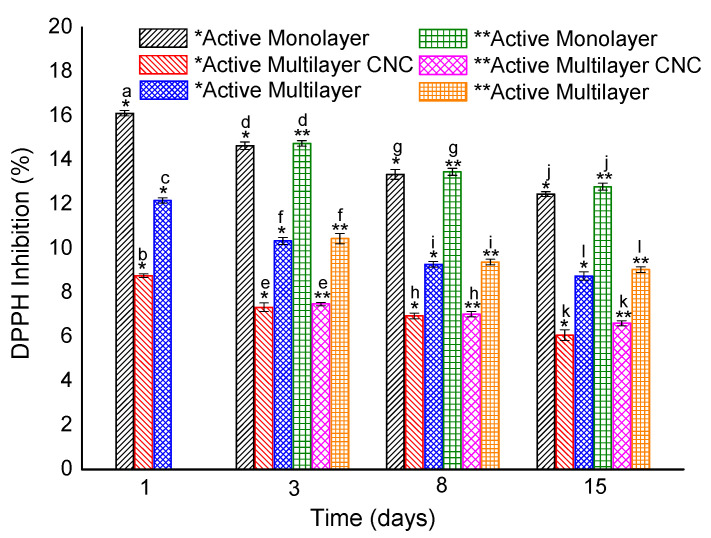
Inhibition percentage (%) of 2,2-diphenyl-1-picrylhydrazyl radical (DPPH) of the active multilayer films with and without cellulose nanocrystal (CNC) coatings for 15 days. * Open system. ** Closed system. a–l: Different superscripts within the same column indicate significant differences among samples (*p* < 0.05).

The antioxidant activity of the materials was studied through the DPPH free radical method, which is an antioxidant assay based on an electron-transfer that produces a violet solution in methanol. Active packaging materials with antioxidant capacity can avoid biochemical reactions caused by light that generates unpleasant aromas and flavors due to oxidation of fats and sugars [58]. Figure 4 and Table 6 show the percent inhibition and the equivalent concentration in micrograms of Trolox per gram of the monolayer and multilayer (with and without CNC) films containing 2.5 wt% OEO + 2.25 wt% ZnONPs in both the open and closed systems for 15 days. As reported in a previous work [21], the antioxidant activity of OEO depends on the secondary metabolites such as, carvacrol, thymol, p-cymene, γ-terpinene, which are responsible of the high DPPH inhibition (91.96%) with respect to other essential oils. Considering the above, the antioxidant activity of the pure OEO into PHBV multilayer and monolayer films decreased due to the low concentration into films and the annealing process that can affect the volatile compounds stability, being sensitive to temperatures above 80 °C [59]. As expected, the active monolayer films showed the major DPPH inhibition in both systems during 15 days compared to the multilayer films, reaching a DPPH inhibition around of 12%, that corresponds to 12.49 and 13.76 (μg eq. Trolox/g sample) for the open and closed system, respectively. The active multilayer films with CNC presented the lowest antioxidant activity of all the samples evaluated, having a DPPH percentage of 6% after 15 days for the open and closed systems with values of 6.25 and 7.04 (μg eq. Trolox/g sample), respectively. The lower antioxidant activity attained in the multilayers could be associated with the presence of the CNC interlayer, which avoided the release of some volatile molecules of OEO in the aqueous medium when the test was performed. Furthermore, during the days of storage, a continuous release of the characteristic volatile compounds was produced. This behavior agrees with the antimicrobial results, where the inhibition of both bacteria was lower for the multilayer with CNC. On the other hand, the active multilayer films without CNC showed a DPPH inhibition of approximately 8.5% at 15 days in open and closed systems with values of 9.00 and 9.29 (μg eq. Trolox/g sample) values, respectively. The DPPH percentage inhibition was significantly different with respect to the multilayer films having the CNC interlayer. This increase could be attributed to the favored release of the OEO molecules to the aqueous medium. For both multilayer films, the behavior in both storage systems (open and closed) did not show significant differences. However, in the different days of evaluation, the multilayer films presented significant differences, showing a reduction of the DPPH inhibition due to the reduced release of the active compounds that are responsible of the antioxidant activity. 

Similar results were reported by Wang et al. [53], who reported that t monolayer films based on curcumin-loaded gelatin prepared by electrospinning showed lower antioxidant activity compared to the pure curcumin whereas multilayer films based on ethylcellulose (EC) nanofibers, as the outer layer, and curcumin-loaded gelatin nanofibers, as the inner layer, showed lower antioxidant activity than the monolayer. Authors concluded that curcumin encapsulated in the gelatin film was well protected by the EC outer layers, which provided a physical barrier for the sustained release of curcumin. Different results were reported by Franco et al. [60] using supercritical carbon dioxide (sc-CO_2_) impregnation technique of α-tocopherol on monolayer and multilayer of PET/PP films performed at 17 MPa and 40 °C. Results of percentage inhibition of the loaded films were similar to pure α-tocopherol after two hours of reaction with DPPH radical. In all cases, the antioxidant activity of the α-tocopherol was preserved after the impregnation with sc-CO_2_. In this way, it is important to point out that the functional activity of the substances and materials depends on the concentration of the active compounds and the process to obtain the materials.

### 3.7. Migration

Migration of nanoparticles is a competitive process that depends on their compatibility with the solid (film) and liquid (food simulant) phases during swelling of the solid phase surface as it comes into contact with the liquid phase [61]. Based on the results obtained in the three food simulants, shown in Table 7, the migration of ZnONPs from the PHA based barrier multilayer film with CNC is definitely influenced by the food simulants. The partial solubility of the biopolymers in each food simulant strongly affects the stability of the ZnONPs embedded into the PHBV matrix. The results revealed greater amounts of zinc released in the acidic solution than in the alcoholic one. Similar results were reported by EFSA [62] where the migration of zinc into 3% (wt/vol) acetic acid was high, up to 17.3 mg/kg, whereas in 10%(vol/vol) ethanol it was up to 80 µg/kg. Furthermore, Ozaki et al. [63] evaluated the migration of zinc from food contact plastics into food simulant such as distillate water, 4% acetic acid, and 20% ethanol, finding that zinc migration was higher in 4% acetic acid due to the higher tendency of ionization. On the other hand, our previous research work evaluated the ZnONPs migration from electrospun active monolayer films of PHA containing 2.5 wt.% OEO + 2.25 wt.% ZnONPs [24], showing a migration value in acidic solution of 6.05 ± 0.81 mg/L and of 0.61 ± 0.1 mg/L in alcoholic solution. In comparison with the migration values of the here-developed active multilayer films, the ZnONPs migration was much lower than that observed for the equivalent monolayer films. These results indicate that the presence of other layers successfully hindered the ZnONPs migration through the whole multilayer. Therefore, the present active multilayer films can be used as an effective antimicrobial packaging for food preservation without affecting food safety. Contrary to the acidic and alcoholic solutions, the fatty simulant, that is, olive oil, led to a high migration of ZnONPs into the fatty simulant. A similar behavior was observed by Heydari-Majd et al. [64], who reported that for a food simulant corresponding to fatty products (95% ethanol), the Zn released was higher. Therefore, according to the new specific migration limit (SML) for soluble ionic Zn, that is, 5 mg/kg food or food simulant, set out by the European Plastics Regulation (EU) 2016/1416 amending [65] and correcting Regulation (EU) 10/2011, that is, 25 mg/kg food or food simulant [66] (see Annex II: Restrictions on materials and articles), all the tested multilayers are supposed to be safe for acidic and alcoholic solutions. However, in the case of the olive oil food simulant, the multilayer slightly exceeded the SML of Zn.

### 3.8. Cytotoxicity

**Figure 5:**
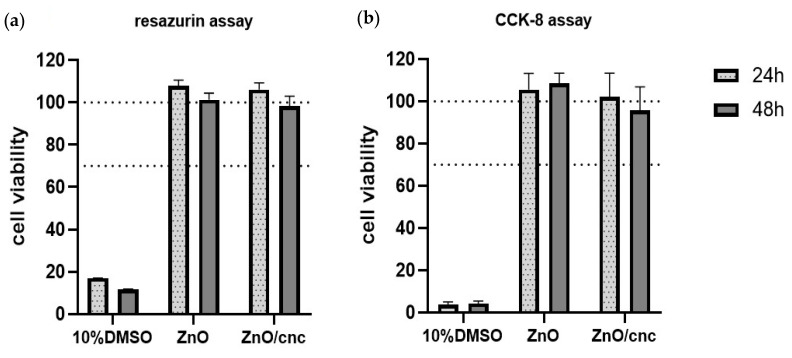
Cytotoxicity assay of the active multilayer films with and without cellulose nanocrystals (CNC) coatings. Cell viability was assessed by the resazurin assay (**a**) and cell counting kit- (CCK-8) assay (**b**) on Caco-2 cells after incubation for 24 and 48 h at 37 °C. Values reported as Mean ± SD (*n* = 8).

The cytotoxicity assay studies the release of components (e.g., ZnONPs) from the materials and their effect on cells viability, determining the biocompatibility and non-toxicity of biodegradable materials, which broadens its application in active food packaging design [67]. In Figure 5, the results of the cytotoxicity assay of the active multilayer films with and without CNC are shown. Cell viability was assessed by the resazurin assay (Figure 5a) and CCK-8 assay (Figure 5b) on Caco-2 cells after incubation for 24 h or 48 h at 37 °C. The results showed that the cell viability was maintained close to 100% throughout the whole experiment, demonstrating the cellular compatibility of the tested multilayer films in indirect contact with the Caco-2 cells. These results show that the films and the substances eventually released from the films are non-toxic to Caco-2 cells. In this regard, Kang et al. [68] studied the cytotoxic effect of three kinds of ZnONPs on human epithelial Caco-2 cells at 24 h exposure. The report concluded that the cytotoxicity of ZnONPs was dose and time dependent and also was influenced by the size, distribution, and nanoparticles intensity. In other study, Apte et al. [69] evaluated the cytotoxicity of multilayer films based on chitosan and alginate, including subsequent cross-linking prepared by layer-by-layer (LbL) technique on viability of human dermal fibroblasts through resazurin assay. The results showed no signs of cytotoxicity in a time frame of 7 days, indicating that cross-linked films made of these biopolymers may be interesting candidates for wound dressings. Frígols et al. [70] also evaluated the cytotoxicity of zinc and graphene oxide (GO) into alginate films cross-linked with Ca^2+^ cations on human keratinocyte HaCaT cells. The results showed that the Zn and GO particles were not cytotoxic for the cell line tested and it was highlighted that zinc release and water sorption/diffusion depended significantly on the type of alginate utilized. In another research work, Ma et al. [71] fabricated poly(ether urethane) (PEU, Biospan^®®^) films by the casting method for the controlled sustained release of the gallium (Ga) or Zn complexes, using polyethylene glycol (PEG) as pore-forming agent. Cell viability of mouse NIH-3 T3 fibroblasts evaluated by alamarBlue™ assay demonstrated no cytotoxicity responses of the Ga- or Zn-complex releasing PEU films. According to the reports mentioned above, it is worthy to highlight that the cytotoxicity of the metallic nanoparticles and other substances does not only depends on their physical and chemical characteristics, but also on the matrix where they are entrapped.

## 4. Conclusions

In response to the constantly evolving food packaging industry requirements, novel research studies are currently focused on the development of environmentally friendly materials with active and barrier properties that are able to extend food shelf life. Active multilayer films with and without CNC coatings in the form of interlayers have been successfully developed in this work, presenting a homogeneous and continuous surface with thickness ranging from 130 to 150 μm. Also, they showed high contact transparency with a slightly yellow appearance. The water and limonene vapor barrier properties of the active multilayer films with the CNC coating were higher than the active multilayer without CNC due to the nanocellulose interlayer. Although the mechanical performance was lower than the neat YPACK210 film, the multilayer films showed balanced mechanical properties. The multilayer films also presented significant inhibition (*R* ≥ 1 and <3) against *S. aureus* and *E. coli* in both storage systems, that is, closed and open, after 15 days. The antioxidant activity tested after 15 days, which is attributed to the presence of OEO, was significantly lower in the active multilayer films with the CNC coating also due to its barrier effect. The migration tests performed on the active multilayer film with CNC revealed that the ZnONPs migration values were lower compared with the monolayer films, being for all food simulants below the SML for Zn, except for olive oil. Lastly, the cytotoxicity assay showed that the cell viability was maintained nearly at 100%, demonstrating the cellular compatibility of the tested active multilayer films in indirect contact with Caco-2 cells.

The novel active multilayer films developed in this study can be regarded as potential candidates for use in the design of sustainable active food packaging. The active and barrier properties of these materials are certainly positive attributes and can be advantageous for food preservation. Since the developed multilayers are based on PHAs they can be applied as compostable or even biodegradable packaging articles, such as food trays or lids. Furthermore, they are partially obtained through the valorization of food waste, contributing to the Circular Bioeconomy progress. Future studies will be focused on analyzing their biodisintegration in both industrial conditions and natural environments as well as their in vivo performance to preserve different foods.

## Figures and Tables

**Table 1:** Color parameters and transparency value of the neat YAPCK210 film and active multilayer films with and without cellulose nanocrystal (CNC) coatings.

Sample	a*	b*	L*	Δ*E*	*T*
YPACK210 film	0.74 ± 0.02 ^a^	−0.41 ± 0.01 ^a^	90.44 ± 0.07 ^a^	-	6.83 ± 0.12 ^a^
Active multilayer with CNC	0.28 ± 0.01 ^b^	1.31 ± 0.02 ^b^	89.81 ± 0.17 ^b^	1.88 ± 0.08 ^a^	4.29 ± 0.15 ^b^
Active multilayer without CNC	0.25 ± 0.03 ^b^	1.42 ± 0.14 ^b^	89.86 ± 0.22 ^b^	1.84 ± 0.19 ^a^	5.94 ± 0.17 ^c^

a–c: Different superscripts within the same column indicate significant differences among samples (*p* < 0.05).

**Table 2:** Thickness and permeance values in terms of water vapor permeance (WVP) and _D_-limonene permeance (LP) of the YPACK210 film and active multilayer films with and without cellulose nanocrystal (CNC) coatings.

Sample	Thickness (mm)	Permeance	
		WVP × 10^11^ (kg·m^−2^·Pa^−1^·s^−1^)	LP × 10^11^ (kg·m^−2^·Pa^−1^·s^−1^)
YPACK210 film 1 layer	0.060	3.22 ± 0.12 ^a^	3.78 ± 0.37 ^a^
YPACK210 1 layer (modeled)	0.140	1.38 ^b^	1.62 ^b^
Active multilayer with CNC *	0.140	0.87 ± 0.92 ^c^	1.36 ± 0.24 ^c^
Active multilayer with CNC **	0.140	0.89 ± 0.50 ^c^	1.39 ± 0.77 ^c^
Active multilayer without CNC	0.131	1.32 ± 0.17 ^b^	1.59 ± 0.38 ^b^

* measured by active interlayer side. ** measured by CNC interlayer side. a–c: Different superscripts within the same column indicate significant differences among samples (*p* < 0.05).

**Table 3:** Mechanical properties in terms of elastic modulus (E), tensile strength at break (σ_b_), elongation at break (ε_b_), and toughness (T) of the YAPCK210 film and active multilayer films with and without cellulose nanocrystal (CNC) coatings in transversal (TD) and machine direction (MD).

Sample	Direction Measure	E (MPa)	$\sigma_{b}\;(\mathrm{MPa})$	$\varepsilon_{b}\;(\%)$	T (mJ/m^3^)
YPACK210 film	TD	2066 ± 284 ^a^	23.1 ± 1.8 ^a^	173 ± 26 ^a^	40.99 ± 8.21 ^a^
Active multilayer with CNC		1491 ± 207 ^b^	20.0 ± 1.4 ^b^	59.1 ± 56 ^b^	11.95 ± 12.3 ^b^
Active multilayer without CNC		1446 ± 190 ^b^	19.2 ± 1.2 ^b^	51.6 ± 45 ^c^	10.36 ± 9.78 ^c^
YPACK210 film	MD	2510 ± 98 ^a^	29.6 ± 1.4 ^a^	76.3 ± 25 ^a^	19.17 ± 6.99 ^a^
Active multilayer with CNC		1828 ± 184 ^b^	23.5 ± 0.7 ^b^	45.6 ± 34 ^b^	9.67 ± 7.67 ^b^
Active multilayer without CNC		1811 ± 79 ^b^	22.28 ± 1.1 ^b^	32.3 ± 17 ^c^	6.87 ± 3.54 ^c^

a–c: Different superscripts within the same column indicate significant differences among samples (*p* < 0.05).

**Table 4:** Antibacterial activity against *Staphylococcus aureus* (*S. aureus*) and *Escherichia coli (E. coli)* of the active multilayer films with and without cellulose nanocrystal (CNC) coatings in the open system for 15 days.

Sample	Bacteria	Day	Control Log (CFU/mL)	Active Log (CFU/mL)	R
Active multilayer with CNC *	*S. aureus*	1	6.95 ± 0.14	5.76 ± 0.09	1.19 ± 0.10
		3	6.90 ± 0.07	5.68 ± 0.08	1.22 ± 0.08
		8	6.89 ± 0.13	5.61 ± 0.15	1.28 ± 0.12
		15	6.91 ± 0.11	5.58 ± 0.12	1.33 ± 0.11
	*E. coli*	1	6.85 ± 0.15	5.72 ± 0.17	1.13 ± 0.16
		3	6.83 ± 0.08	5.64 ± 0.09	1.19 ± 0.07
		8	6.82 ± 0.15	5.60 ± 0.14	1.22 ± 0.13
		15	6.83 ± 0.14	5.57 ± 0.12	1.26 ± 0.11
Active multilayer with CNC **	*S. aureus*	1	6.95 ± 0.14	6.04 ± 0.12	0.91 ± 0.10
		3	6.90 ± 0.07	5.92 ± 0.08	0.98 ± 0.09
		8	6.89 ± 0.13	5.86 ± 0.11	1.03 ± 0.12
		15	6.91 ± 0.11	5.83 ± 0.09	1.08 ± 0.10
	*E. coli*	1	6.85 ± 0.15	5.99 ± 0.13	0.86 ± 0.13
		3	6.83 ± 0.08	5.92 ± 0.09	0.91 ± 0.08
		8	6.82 ± 0.15	5.83 ± 0.14	0.99 ± 0.12
		15	6.83 ± 0.14	5.79 ± 0.11	1.04 ± 0.13
Active multilayer without CNC	*S. aureus*	1	6.95 ± 0.14	5.74 ± 0.15	1.21 ± 0.17
		3	6.90 ± 0.07	5.66 ± 0.09	1.24 ± 0.09
		8	6.89 ± 0.13	5.59 ± 0.12	1.30 ± 0.15
		15	6.91 ± 0.11	5.56 ± 0.08	1.35 ± 0.10
	*E. coli*	1	6.85 ± 0.15	5.69 ± 0.12	1.16 ± 0.08
		3	6.83 ± 0.08	5.63 ± 0.09	1.20 ± 0.10
		8	6.82 ± 0.15	5.57 ± 0.13	1.25 ± 0.14
		15	6.83 ± 0.14	5.56 ± 0.11	1.27 ± 0.12

* measured by active interlayer side. ** measured by CNC interlayer side. Antibacterial activity was quantified as the test surface reduction (R) from the colony forming units (CFU)/mL.

**Table 5:** Antibacterial activity against *Staphylococcus aureus* (*S. aureus*) and *Escherichia coli (E. coli)* of the active multilayer films with and without cellulose nanocrystal (CNC) coatings in the closed system for 15 days.

Sample	Bacteria	Day	Control Log (CFU/mL)	Multilayer Log (CFU/mL)	R
Active multilayer with CNC *	*S. aureus*	1	6.95 ± 0.14	5.75 ± 0.11	1.20 ± 0.09
		3	6.90 ± 0.07	5.66 ± 0.08	1.24 ± 0.07
		8	6.89 ± 0.13	5.59 ± 0.12	1.30 ± 0.11
		15	6.91 ± 0.11	5.57 ± 0.08	1.34 ± 0.07
	*E. coli*	1	6.85 ± 0.15	5.70 ± 0.14	1.15 ± 0.13
		3	6.83 ± 0.08	5.62 ± 0.09	1.21 ± 0.08
		8	6.82 ± 0.15	5.58 ± 0.14	1.24 ± 0.11
		15	6.83 ± 0.14	5.54 ± 0.11	1.29 ± 0.09
Active multilayer with CNC **	*S. aureus*	1	6.95 ± 0.14	6.02 ± 0.16	0.93 ± 0.15
		3	6.90 ± 0.07	5.90 ± 0.07	1.00 ± 0.08
		8	6.89 ± 0.13	5.83 ± 0.18	1.06 ± 0.16
		15	6.91 ± 0.11	5.81 ± 0.09	1.10 ± 0.09
	*E. coli*	1	6.85 ± 0.15	5.96 ± 0.13	0.89 ± 0.12
		3	6.83 ± 0.08	5.90 ± 0.09	0.93 ± 0.11
		8	6.82 ± 0.15	5.81 ± 0.12	1.01 ± 0.14
		15	6.83 ± 0.14	5.77 ± 0.15	1.06 ± 0.12
Active multilayer without CNC	*S. aureus*	1	6.95 ± 0.14	5.72 ± 0.09	1.23 ± 0.10
		3	6.90 ± 0.07	5.64 ± 0.08	1.26 ± 0.07
		8	6.89 ± 0.13	5.57 ± 0.11	1.32 ± 0.11
		15	6.91 ± 0.11	5.54 ± 0.10	1.37 ± 0.09
	*E. coli*	1	6.85 ± 0.15	5.67 ± 0.16	1.18 ± 0.18
		3	6.83 ± 0.08	5.61 ± 0.07	1.22 ± 0.08
		8	6.82 ± 0.15	5.54 ± 0.14	1.28 ± 0.14
		15	6.83 ± 0.14	5.53 ± 0.19	1.30 ± 0.17

* measured by active interlayer side. ** measured by CNC interlayer side. Antibacterial activity was quantified as the test surface reduction (R) from the colony forming units (CFU)/mL.

**Table 6:** Concentration (μg eq. Trolox/g sample) of 2,2-diphenyl-1-picrylhydrazyl radical (DPPH) of the active multilayer films with and without cellulose nanocrystal (CNC) coatings in the open a closed system for 15 days.

Sample	Day	Open System	Closed System
		(μg eq. Trolox/g Sample)	(μg eq. Trolox/g Sample)
Active monolayer	1	17.44 ± 0.14 ^a^	---
	3	15.94 ± 0.10 ^b,A^	15.98 ± 0.19 ^a,A^
	8	14.28 ± 0.17 ^c,B^	14.42 ± 0.02 ^b,B^
	15	12.49 ± 0.08 ^d,C^	13.76 ± 0.12 ^c,D^
Active multilayer with CNC	1	9.11 ± 0.09 ^e^	---
	3	7.74 ± 0.03 ^f,E^	8.14 ± 0.02 ^d,F^
	8	7.42 ± 0.01 ^f,G^	7.44 ± 0.01 ^e,G^
	15	6.25 ± 0.02 ^g,H^	7.04 ± 0.12 ^e,I^
Active multilayer without CNC	1	12.66 ± 0.12 ^h^	---
	3	10.90 ± 0.02 ^i,J^	11.01 ± 0.06 ^f,J^
	8	9.67 ± 0.03 ^j,K^	10.07 ± 0.03 ^g,L^
	15	9.00 ± 0.09 ^k,M^	9.29 ± 0.04 ^h,M^

a–k: Different superscripts within the same column indicate significant differences among samples (*p* < 0.05). A–M: Different superscripts within the same row indicate significant differences among samples (*p* < 0.05).

**Table 7:** Determination of the amount of zinc (Zn) migrated from the active multilayer films with cellulose nanocrystal (CNC) coating into different food simulants after an incubation period of 10 days at 40 °C.

Sample	Food Simulants					
	10% (vol/vol) Aqueous Ethanol		3% (wt/vol) Aqueous Acetic Acid		Olive Oil	
	Zn (mg/L)	Zn (mg/dm^2^)	Zn (mg/L)	Zn (mg/dm^2^)	Zn (mg/L)	Zn (mg/dm^2^)
Active multilayer with CNC	0.051 ± 0.019	0.00089 ± 0.00021	0.185 ± 0.401	0.0032 ± 0.0051	26.662 ± 11.303	0.426 ± 0.142
